# *Bifidobacterium longum* JBLC-141 alleviates hypobaric hypoxia-induced intestinal barrier damage by attenuating inflammatory responses and oxidative stress

**DOI:** 10.3389/fmicb.2024.1501999

**Published:** 2024-12-17

**Authors:** Xiang-Yang Li, Jin Shang, Xiao-Juan Wang, Hui-Ping Ma, Long-Fei Ren, Lei Zhang

**Affiliations:** ^1^The First Clinical Medical College, Lanzhou University, Lanzhou, Gansu, China; ^2^Department of General Surgery, The First Hospital of Lanzhou University, Lanzhou, Gansu, China; ^3^Laboratory of Biological Therapy and Regenerative Medicine Transformation Gansu Province, The First Hospital of Lanzhou University, Lanzhou, Gansu, China; ^4^Pharmacy Department, The 940 Hospital of Joint Logistics Support, PLA, Lanzhou, Gansu, China; ^5^National Clinical key Specialty of General Surgery, The First Hospital of Lanzhou University, Lanzhou, Gansu, China; ^6^Clinical Research Center for General Surgery of Gansu Province, Lanzhou, Gansu, China

**Keywords:** *Bifidobacterium longum*, hypobaric hypoxia, oxidative stress, intestinal barrier, intestinal flora

## Abstract

Hypobaric hypoxia exposure occurs at high altitudes, including plateaus, and affects normal intestinal function and microbiota composition. Exposure induces an intestinal inflammatory response and oxidative stress injury, ultimately disrupting intestinal homeostasis and causing barrier damage. Thus, due to its anti-inflammatory, antioxidative, and intestinal microbiota-regulating properties, *Bifidobacterium longum* is a potentially effective probiotic intervention to protect the intestinal barrier during low-pressure hypoxia on plateaus. However, its mechanism of action is not fully defined. In this study, we investigate the mechanism by which *B. longum* intervenes in intestinal barrier damage caused by plateau low-pressure hypoxia. To this end, an *in vivo* model is established by exposing rats to a simulated low-pressure hypoxic plateau environment. The experimental rats were subsequently supplemented with a *B. longum* strain (JBLC-141) extracted from the feces of healthy adults in Bama, Guangxi. *B. longum* JBLC-141 mitigates the effects of plateau low-pressure hypoxia on the rat intestinal barrier. This is achieved by activating the intestinal Kelch-like ECH-associated protein 1 (KEAP1)/nuclear factor erythroid 2-related factor 2 (NRF2) pathway, alleviating plateau hypoxia-induced intestinal oxidative stress injury. *B. longum* JBLC-141 also attenuates the inflammatory response and upregulates the expression of the tight junction proteins claudin-1, occludin, and zonula occludens-1. Furthermore, it reduces intestinal permeability, effectively ameliorating and repairing the barrier histological damage induced by the plateau low-pressure hypoxic environment. In addition, *B. longum* JBLC-141 positively regulates the intestinal microbiota, increasing the relative abundance of beneficial bacteria while reducing that of pathogenic bacteria and maintaining intestinal flora homeostasis in rats.

## 1 Introduction

Globally, approximately 81.6 million people live at altitudes >2,500 m (Tremblay and Ainslie, 2021). Residing at high altitudes can adversely affect human physical and mental health (Jia et al., 2020). In particular, the associated low-pressure hypoxia is an important health factor (Li et al., 2024; McKenna et al., 2022), disrupting gastrointestinal hormone and digestive fluid secretion and inhibiting gastrointestinal peristalsis (Li et al., 2024). Consequently, individuals traveling from plains to hypoxic plateaus are prone to acute digestive stress, including nausea, vomiting, flatulence, epigastric discomfort, severe acidosis, and diarrhea (Davis and Hackett, 2017).

Exposure to low-pressure hypoxia at high altitudes decreases visceral perfusion and blood oxygen levels (König et al., 2016; McKenna et al., 2022). Hypoxia also leads to oxidative stress (Wang et al., 2015) and promotes pro-inflammatory cascades (Watson et al., 2015). The subsequent reduction in tight junction protein expression by enterocytes disrupts the intestinal mechanical barrier and increases intestinal permeability (Ni et al., 2012; Schüller et al., 2009), leading to intestinal microecological imbalances (Luo et al., 2017; Zhou et al., 2011). The associated increase in bacterial translocation and decrease in occludin (OCLN) expression facilitates the entry of hazardous substances, such as lipopolysaccharide (LPS), into the bloodstream (Luo et al., 2017). Plateau hypoxia also alters the intestinal microflora composition, which may cause intestinal mucosal damage (Sun et al., 2020; Xu et al., 2014; Zhang J. et al., 2018; Zhang W. et al., 2018). However, there are no effective methods for preventing and treating intestinal barrier dysfunction or gut microbiome disorder in hypoxic environments.

*Bifidobacterium longum*, one of the most abundant species of the genus *Bifidobacterium*, modulates oxidative stress by enhancing antioxidant activity and regulating the production and accumulation of reactive oxygen species (ROS), alleviating symptoms of oxidative damage to the intestinal barrier. Treatment of dextrose sodium sulfate-induced colitis in mice with *B. longum* reduces eosinophil peroxidase levels (Abrantes et al., 2020; Zhang et al., 2022a); similar antioxidant effects are elicited by *B. longum* metabolites (Yan et al., 2020; Zhang et al., 2023). Without altering cell viability, the *B. longum* culture supernatant can increase the intracellular antioxidant capacity, enhance intracellular catalase activity, and reduce nicotinamide adenine dinucleotide phosphate (NADPH) oxidase activation (Wang et al., 2021). Additionally, its anti-inflammatory metabolite indole-3-lactic acid (ILA) upregulates cytochrome P450 1A1 (*CYP1A1*) mRNA expression—an aryl hydroxyl receptor (AHR) target gene. ILA also upregulates the nuclear factor erythroid 2-related factor 2 (NRF2) target genes, glutathione reductase 2 (*GPX2*), superoxide dismutase 2 (*SOD2*), and NAD(P)H dehydrogenase (*NQO1*) (Ehrlich et al., 2020). These effects protect intestinal epithelial cells *in vitro* by activating the AHR and nuclear factor erythroid 2-related factor 2 (NRF2) pathways (Ehrlich et al., 2020).

*Bifidobacterium longum* short-chain fatty acid (SCFA) metabolites also increase connexin abundance in intestinal epithelial cells by enhancing mucin 2 (*MUC2*) mRNA expression and activating the AMP-activated protein kinase (AMPK) pathway (Ghosh et al., 2021). Additionally, *B. longum* decreases the abundance of pathogenic bacteria while increasing that of beneficial bacteria in the gastrointestinal tract (Yan et al., 2020; Yun et al., 2017). This helps modulate diseases related to intestinal barrier damage caused by intestinal microecological dysregulation.

The aim of this study is to investigate whether the probiotic *B. longum* strain JBLC-141 can ameliorate hypoxia-induced intestinal mucosal barrier damage, inflammatory responses, and intestinal flora dysregulation. We hypothesize that *B. longum* alleviates the intestinal barrier damage induced by plateau hypobaric hypoxia by protecting the intestinal barrier and regulating the intestinal flora. To test this hypothesis, we have established a rat model of hypoxia pretreated with *B. longum* JBLC-141.

## 2 Materials and methods

### 2.1 Bacterial strains, reagents, and equipment

*Bifidobacterium longum* (Item No.: JBLC-141) was purchased from Shandong Zhongke Jiayi Bioengineering Co. Ltd. (Shandong, China). Pentobarbital sodium solution, sodium chloride (NaCl) solution (saline), sterilized water, suture needles, syringes, and gauze were purchased from the First Hospital of Lanzhou University, China. Tissue scissors, hemostatic forceps, forceps, arterial clips, and other surgical instruments were provided by the Laboratory of the First Hospital of Lanzhou University, China.

### 2.2 Laboratory animals and sample collection

All animal protocols were approved by the Ethics Committee of the First Hospital of Lanzhou University (approval number: LDYYLL2024-537).

Thirty-two 6-week-old male Sprague–Dawley rats were purchased from Lanzhou Veterinary Research Institute. The rats were housed in a clean animal rearing room at 20–25°C, 20–40% humidity, under a 12-h light/dark cycle with free access to food and water. After a one-week acclimatization period, the rats were randomly divided into four groups (*n* = 8/group): normoxia plus saline (SS), normoxia plus probiotic (SB), hypoxia plus saline (LS), and hypoxia plus probiotic (LB). Each group was prophylactically administered 2 mL of 0.9% saline or an equal volume of a solution containing 1 × 10^11^ CFU/mL *B. longum* via intragastric administration for five days. Twenty-four hours after the last administration, the LS and LB groups were placed in the plateau low-pressure oxygen simulation chamber. This chamber increased to an altitude of 6,000 m (oxygen [O_2_] content: 13%, barometric pressure: 354 mmHg) at an average speed of ~5 m/s to simulate continuous highland hypoxia for six days. During continuous exposure to hypoxia, the cabin was opened once daily for 30 min to provide food and water, operate gavage, measure the body weight, and collect feces on the day of end of the experiment. The SS and SB groups were maintained in a normoxia environment outside the chamber of the Basic Medical Laboratory of the 940th Hospital of the United Logistics Force (Lanzhou, 1,500 m above sea level, air pressure: 635 mmHg).

On Day 6 of exposure in the plateau hypoxia simulation chamber, the rats were anesthetized with sodium pentobarbital solution (1%, 50 mg/kg); blood was collected from the abdominal aorta, from which serum was prepared and stored at −80°C. Fresh colon tissues and the intestinal contents were collected from each group of rats after they were euthanized, and half of the colon tissue samples were clipped, frozen rapidly in liquid nitrogen, and stored at −80°C. The other half of the samples were fixed by immersion in a 4% paraformaldehyde solution and stored for further examination. To minimize pain and distress as much as possible in the rats, all samples were collected under anesthesia induced using a sodium pentobarbital solution, which ended in their death.

### 2.3 Analysis of serum inflammatory cytokines and intestinal permeability

Serum levels of interleukin (IL)-6, tumor necrosis factor *α* (TNF-α), and IL-10 were detected using enzyme-linked immunosorbent assay (ELISA) kits (Boster, Wuhan, China). Serum levels of diamine oxidase (DAO) and d-lactic acid (DLA) were detected using biochemical analysis kits (Elabscience, Wuhan, China), following the manufacturer’s instructions.

### 2.4 Analysis of intestinal redox homeostasis and barrier proteins

The malondialdehyde (MDA) and superoxide dismutase (SOD) contents of colonic tissues were determined using appropriate commercial kits according to the manufacturer’s instructions (Elabscience, Wuhan, China). OCLN, claudin-1 (CLDN1), and zonula occludens-1 (ZO-1) levels in the colon tissues were determined using analysis kits according to the manufacturer’s instructions (Meilian, Shanghai, China).

### 2.5 Histological and apoptosis analyses

Rat colonic samples were fixed in a 4% paraformaldehyde solution, embedded in paraffin, cut into 4-μm slices, and stained with hematoxylin and eosin (H&E). Images were acquired for analysis under a positive light microscope (Nikon Eclipse E100; Nikon, Tokyo, Japan) at 100× and 200× magnification.

The terminal deoxynucleotidyl transferase deoxyuridine dUTP nick-end labeling (TUNEL) method was used to detect apoptosis of intestinal tissue. Fixed intestinal tissues were routinely paraffin-embedded, sectioned, and dewaxed; a proteinase K working solution (proteinase K storage solution diluted with phosphate-buffered saline [PBS] 1:9) was applied drop-wise and incubated for 30 min at 37°C. Subsequently, the tissues were washed with PBS (pH 7.4) thrice for 5 min each, shaken dry, and membrane-breaking working solution was added drop-wise. They were incubated for 20 min and washed with PBS thrice for 5 min each. Proteinase K working solution was added for 20 min at room temperature. The sections were subsequently incubated for 2 h at 37°C, washed with PBS thrice for 5 min each, and incubated in 3% hydrogen peroxide solution prepared with methanol (hydrogen peroxide to methanol = 1:9) for 15 min in the dark. The sections were washed with PBS thrice, dried, and an appropriate amount of reagent 3 (converter-PP) was added (converter-POD), followed by incubation at 37°C for 30 min and washing thrice with PBS for 5 min each. The sections were shaken dry, freshly prepared 3,3′-diaminobenzidine (DAB) color development solution was applied under the microscope to control the color development time, and the sections were rinsed with tap water to terminate the color development. This was followed by re-staining with Harris hematoxylin for approximately 3 min, washing with tap water, and applying 1% hydrochloric acid alcohol for a few seconds. The sections were rinsed with tap water, treated with ammonia, rinsed in tap water, and washed in ammonia water again to restore the blue color. Finally, the sections were dehydrated, dried, sealed, and scanned using a digital section scanner to collect images and data.

Caspase 3 Activity (Elabscience, Wuhan, China) and B-cell lymphoma 2 (BCL-2) (Meilian, Shanghai, China) levels in the colonic tissues were determined using analysis kits according to the manufacturer’s instructions.

### 2.6 Western blot analysis

Total protein was extracted from rat colonic tissue samples using abrasive beads and radioimmunoprecipitation assay (RIPA) lysis buffer. Protein concentration was measured using a bicinchoninic acid (BCA) assay kit (both from Servicebio, Wuhan, China). Proteins were electrophoresed using 10% sodium dodecyl sulfate-polyacrylamide gel electrophoresis and transferred onto a polyvinylidene fluoride membrane. After incubation with skim milk to block non-specific binding, the membrane was incubated with primary antibodies at 4°C overnight. The membrane was washed with Tris-buffered saline (TBST), incubated with a secondary antibody for 30 min at room temperature, and washed once with TBST. Protein bands were visualized using the enhanced chemiluminescence (ECL) luminescent liquid combo kit (Servicebio), and data were analyzed using the AIWBwell analysis software (Servicebio). The antibody sources, catalog numbers, and dilutions are listed in Supplementary Table S1.

### 2.7 Extraction, sequencing, and analysis of intestinal flora DNA

Microbial DNA was extracted from feces using the TGuide S96 magnetic soil/stool DNA kit (Tiangen Biotech Co., Ltd., Beijing) according to the manufacturer’s instructions. The 338F: 5’ACTCCTACGGGAGGCAGCA-3′ and 806R: 5’GGACTACHVGGGTWTCTAAT-3′ universal primer set was used to amplify the V3–V4 region of the *16S* rRNA gene from the genomic DNA extracted from each sample, with the Illumina Novaseq 6,000 sequencing system (Illumina, Santiago CA, USA). Bioinformatics analysis was performed using the BMK Cloud (Biomarker Technologies Co., Ltd., Beijing, China). Based on the quality of a single nucleotide, raw data were filtered using Trimmomatic (version 0.33).

Primer sequences were identified and removed using the Cutadapt software (version 1.9.1). Paired-end reads obtained from the previous steps were assembled using USEARCH (version 10), followed by chimera removal using UCHIME (version 8.1). Sequences with similarity ≥97% were clustered into the same operational taxonomic unit (OTU); OTUs with re-abundance <0.005% were filtered. Taxonomic annotation of the OTUs was performed based on the naïve Bayes classifier in QIIME2 using the SILVA database (release 132) with a confidence threshold of 70%. Alpha diversity was calculated and displayed using QIIME2 and R software programs, respectively.

Beta diversity was determined using QIIME to evaluate the degree of similarity between the microbial communities from different samples. Principal coordinate analysis (PCoA), heat maps, unweighted pair group method with arithmetic mean (UPGMA), and non-metric multidimensional scaling were used to analyze the beta diversity. Furthermore, linear discriminant analysis (LDA) effect size (LEfSe) was used to identify significant taxonomic differences among groups. A logarithmic LDA score of 3.5 was set as the threshold for discriminative features. To explore the dissimilarities in the microbiome among different factors, redundancy analysis was performed with the R software using the “vegan” package.

### 2.8 Statistical analysis

The experimental data were statistically analyzed and graphed using GraphPad Prism version 10 (GraphPad Software, Inc., San Diego, CA, USA), and data were expressed as mean ± standard error of the mean. Comparisons between multiple groups of quantitative data were performed using a one-way analysis of variance (ANOVA). Results with a *p* < 0.05 were considered statistically significant.

## 3 Results

### 3.1 JBLC-141 reduces intestinal injury and inflammation and enhances intestinal barrier function in rats in a plateau hypoxic environment

**Figure 1:**
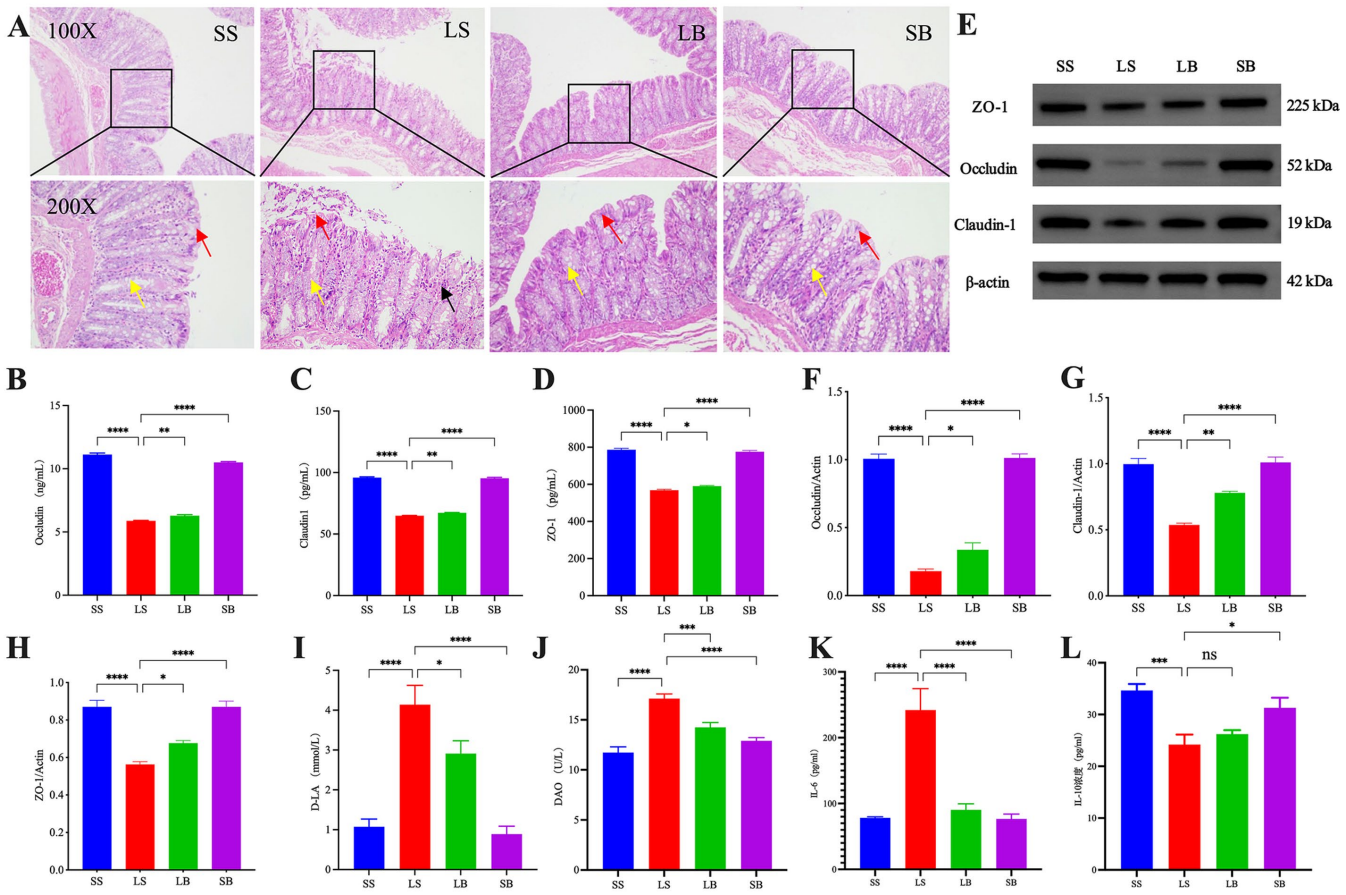
*Bifidobacterium longum* JBLC-141 pretreatment ameliorates intestinal barrier damage in rats under simulated plateau hypobaric hypoxic conditions. **(A)** Hematoxylin and eosin (H&E) staining of intestinal colonic tissue histopathology of each rat group (100×/200×). **(B–D)** Assay kit detection of occludin (OCLN), claudin-1 (CLDN1), and zonula occludens-1 (ZO-1) in colonic tissues. **(E–H)** Western blot analysis of OCLN, CLDN1, and ZO-1 protein expression in colon tissue. **(I,J)** Assay kit detection of serum concentrations of indicators of intestinal function, d-lactic acid (DLA), and diamine oxidase (DAO). **(K,L)** Enzyme-linked immunosorbent assay (ELISA) detection of serum levels of inflammatory factors, interleukin-6 (IL-6) and IL-10. Not significant (ns) *p* > 0.05, *p < 0.05, **p < 0.01, ***p < 0.001, and ****p < 0.0001. Red, yellow, and black arrows in a: chyme detached epithelial, goblet, and inflammatory cells, respectively, of the mucosal layer.

H&E staining of the rat colonic tissues (Figure 1A) showed that the SS group had normal intestinal tissue structure. The epithelial cells of the mucosal layer were tightly arranged, with no substantial erosion or detachment; numerous crypts and goblet cells were observed. Furthermore, the tissue was not substantially infiltrated by inflammatory cells. In contrast, the intestinal tissue of the LS group exhibited an abnormal structure with eroded and some detached epithelial cells in the mucosal layer. In addition, the crypts were disordered, with reduced goblet cells and increased inflammatory cells in the mucosal layer. The LB group exhibited a normal intestinal tissue structure with tightly arranged epithelial cells in the mucosal layer and structurally normal crypts. Moreover, the goblet cells were abundant, and no obvious inflammatory cell infiltration. The SB group also showed a normal intestinal tissue structure, with intact epithelial cells in the mucosal layer and numerous goblet cells, comparable to that of the SS group.

Serum IL-6 levels were significantly higher (*p* < 0.0001; Figure 1K) in the LS group than in the SS group, whereas that of IL-10 was significantly lower (*p* < 0.001; Figure 1L). Pretreatment of LB group with JBLC-141 significantly decreased IL-6 levels (*p* < 0.0001) and slightly increased IL-10 (*p* > 0.05) compared with the LS group.

ELISA results revealed that colonic tissue levels of OCLN, CLDN1, and ZO-1 were significantly lower in the LS group than in the SS group (all *p* < 0.0001; Figures 1B–D). JBLC141 pretreatment significantly increased the expression of these proteins in the LB group (OCLN: *p* < 0.01, CLDN1: *p* < 0.01, and ZO-1: *p* < 0.05, respectively) compared with the LS group. These results were corroborated by western blotting (Figures 1E–H). Furthermore, the protein expression level analysis indicated good intestinal barrier repair.

The detection of rat serum DAO and DLA levels (Figures 1I,J) showed significantly higher levels in the LB group than in the SS group (both *p* < 0.0001). Their levels were significantly lower in the JBLC-141-pretreated LB group than in the LS group (*p* < 0.05 and *p* < 0.001, respectively).

### 3.2 JBLC-141 inhibits intestinal cell apoptosis

**Figure 2:**
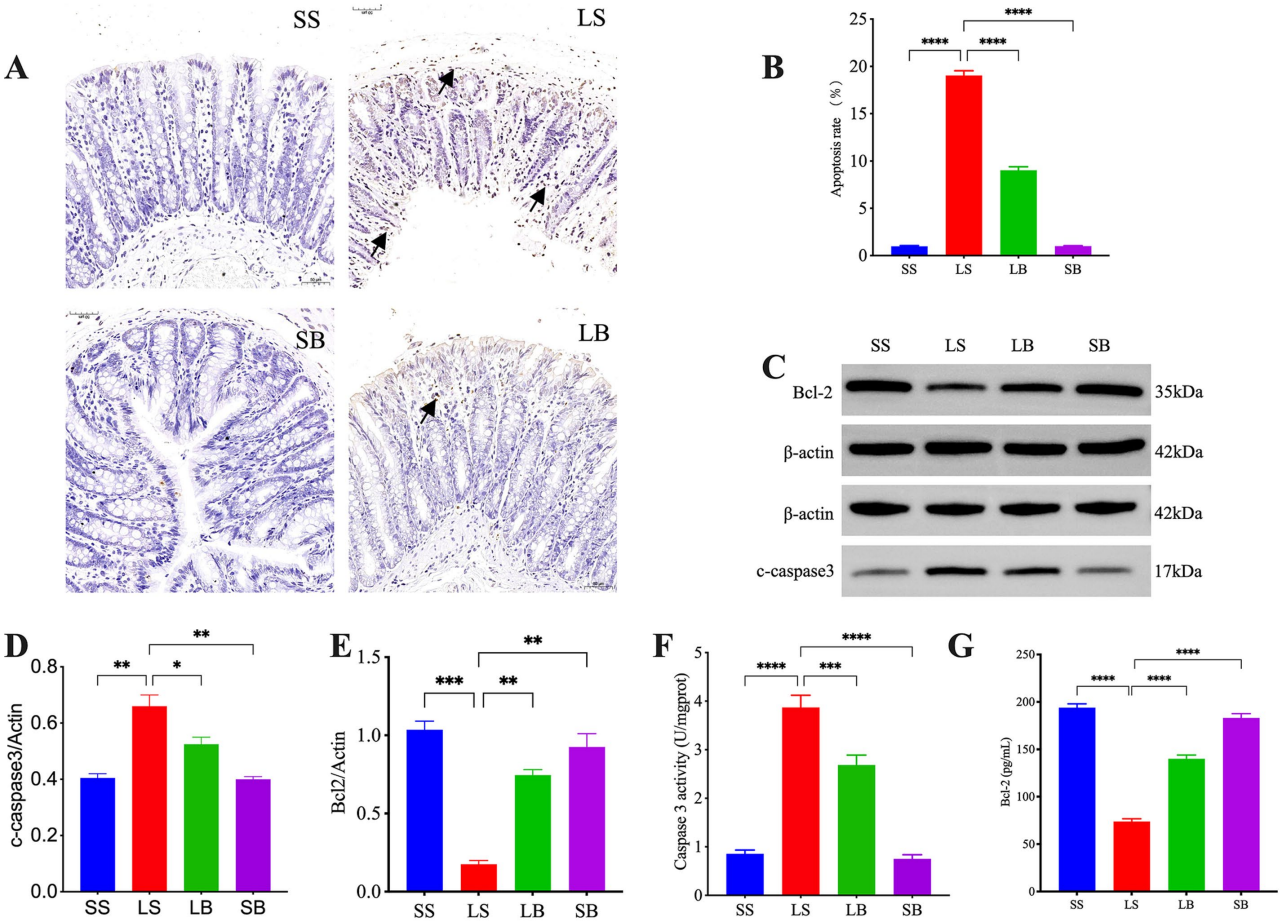
*Bifidobacterium longum* JBLC-141 pretreatment inhibits apoptosis of intestinal epithelial cells. **(A,B)** Terminal deoxynucleotidyl transferase (TdT) deoxyuridine dUTP nick-end labeling (TUNEL) detection of intestinal epithelial cell apoptosis (50 μm). **(C–E)** Western blot analysis of relative expression levels of Cleaved Caspase 3 and B-cell CLL/lymphoma 2 (BCL-2) proteins in colon tissues. **(F,G)** Assay kit detection of Caspase 3 Activity and BCL-2 levels in colon tissue specimens. Not significant (ns) *p* > 0.05, *p < 0.05, **p < 0.01, ***p < 0.001, and ****p < 0.0001. Black arrows in **(A)**: tan apoptotic signal.

TUNEL analysis of intestinal tissue apoptosis (Figures 2A,B) showed no obvious apoptotic cells in the SS group. In contrast, numerous brownish-yellow apoptotic signals were observed in the mucosal layer of the LS group. Meanwhile, significantly fewer apoptotic signals were detected in the LB group than in the LS group. The colonic structure of the SB group was comparable to that of the SS group, with no apoptotic signals observed.

Detection of Caspase 3 Activity and B-cell lymphoma 2 (BCL-2) levels in the colonic tissues (Figures 2F,G) showed that the Caspase 3 Activity level in the LS group was significantly higher than in the SS group (*p* < 0.0001). Their abundance was significantly lower in the LB group than in the LS group (*p* < 0.001). The BCL-2 level was significantly lower in the LS and LB groups (both *p* < 0.0001) than in the SS group and significantly higher in the LB group than in the LS group (*p* < 0.0001). These results were corroborated by western blot analysis (Figures 2C–E).

### 3.3 JBLC-141 activates the intestinal Kelch-like ECH-associated protein 1 (KEAP1)/NRF2 pathway to alleviate oxidative stress injury in plateau hypoxic rats

**Figure 3:**
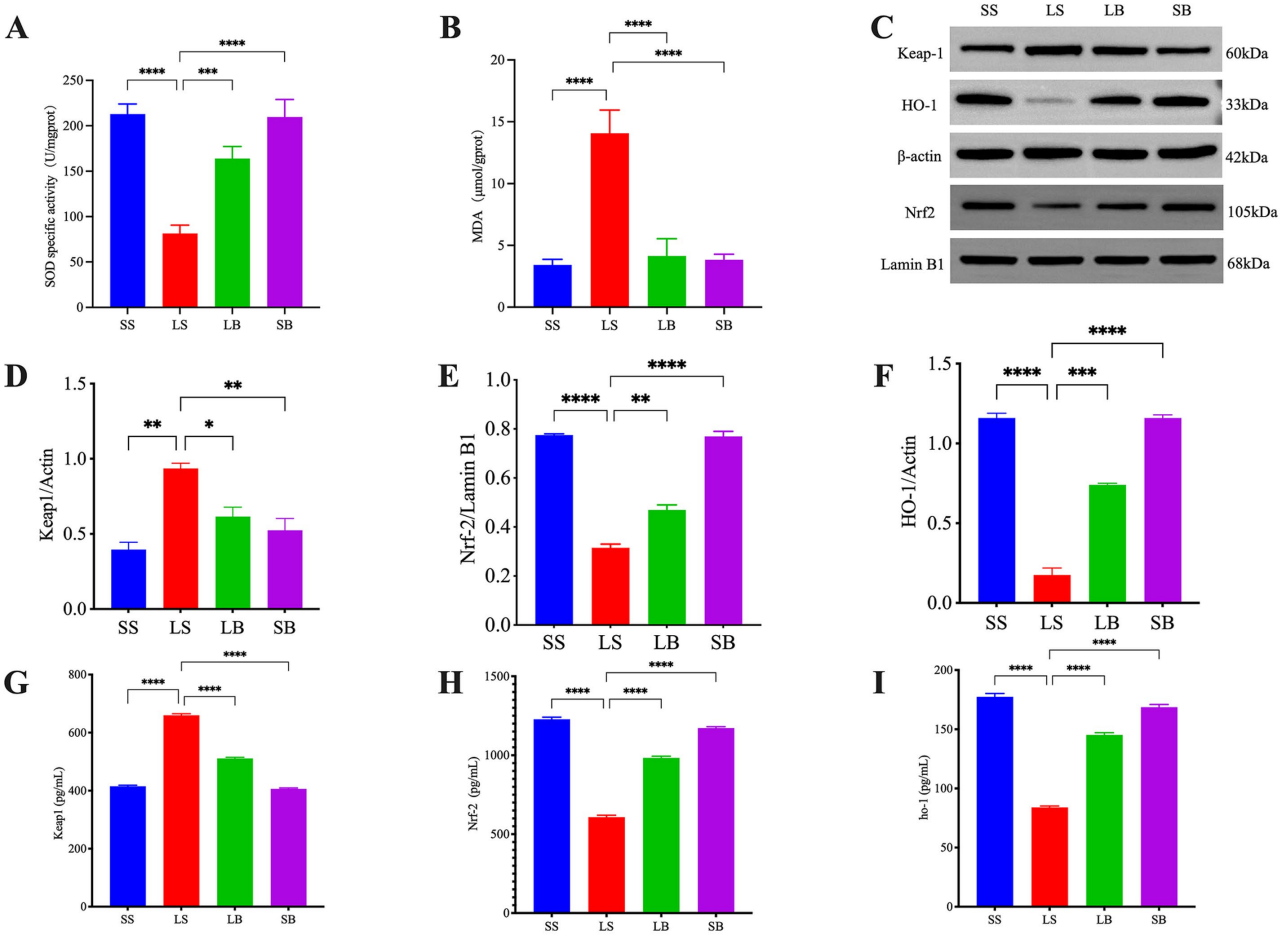
*Bifidobacterium longum* JBLC-141 pretreatment alleviates intestinal oxidative stress injury. **(A,B)** Assay kit detection of superoxide dismutase (SOD) and malondialdehyde (MDA), indicators of oxidative stress, in colon tissues. **(C–F)** Western blot detection of relative expression levels of KEAP1, NRF2, and HO-1 proteins in colon tissues. **(G–I)** Assay kit detection of Kelch-like ECH-associated protein 1 (KEAP1), nuclear factor erythroid 2-related factor 2 (NRF2), and heme oxygenase-1 (HO-1) in colon tissue specimens. Not significant (ns) *p* > 0.05, *p < 0.05, **p < 0.01, ***p < 0.001, and ****p < 0.0001.

To determine the antioxidant effects of JBLC-141, MDA and SOD levels were detected in hypoxic rat colonic tissues. A significantly higher MDA level (*p* < 0.0001; Figure 3A) and lower SOD activity (*p* < 0.0001; Figure 3B) was observed in the LS group than in the SS group. JBLC-141 pretreatment reversed in the LB group (MDA, *p* < 0.0001; SOD, *p* < 0.001).

Western blotting revealed significantly higher KEAP-1 levels in the colonic tissues of the LS group compared with the SS group (*p* < 0.01) and significantly lower levels of NRF2 (*p* < 0.0001; Figures 3C–F) and heme oxygenase 1 (HO-1; *p* < 0.0001). These changes in KEAP-1 (*p* < 0.05), NRF2 (*p* < 0.05), and HO-1 (*p* < 0.001) were significantly more suppressed in the LB group than in the LS group after JBLC-141 pretreatment. These results were corroborated by biochemical assays (Figures 3G–I).

### 3.4 JBLC-14 ameliorates intestinal damage by regulating intestinal flora in plateau hypoxic rats

**Figure 4:**
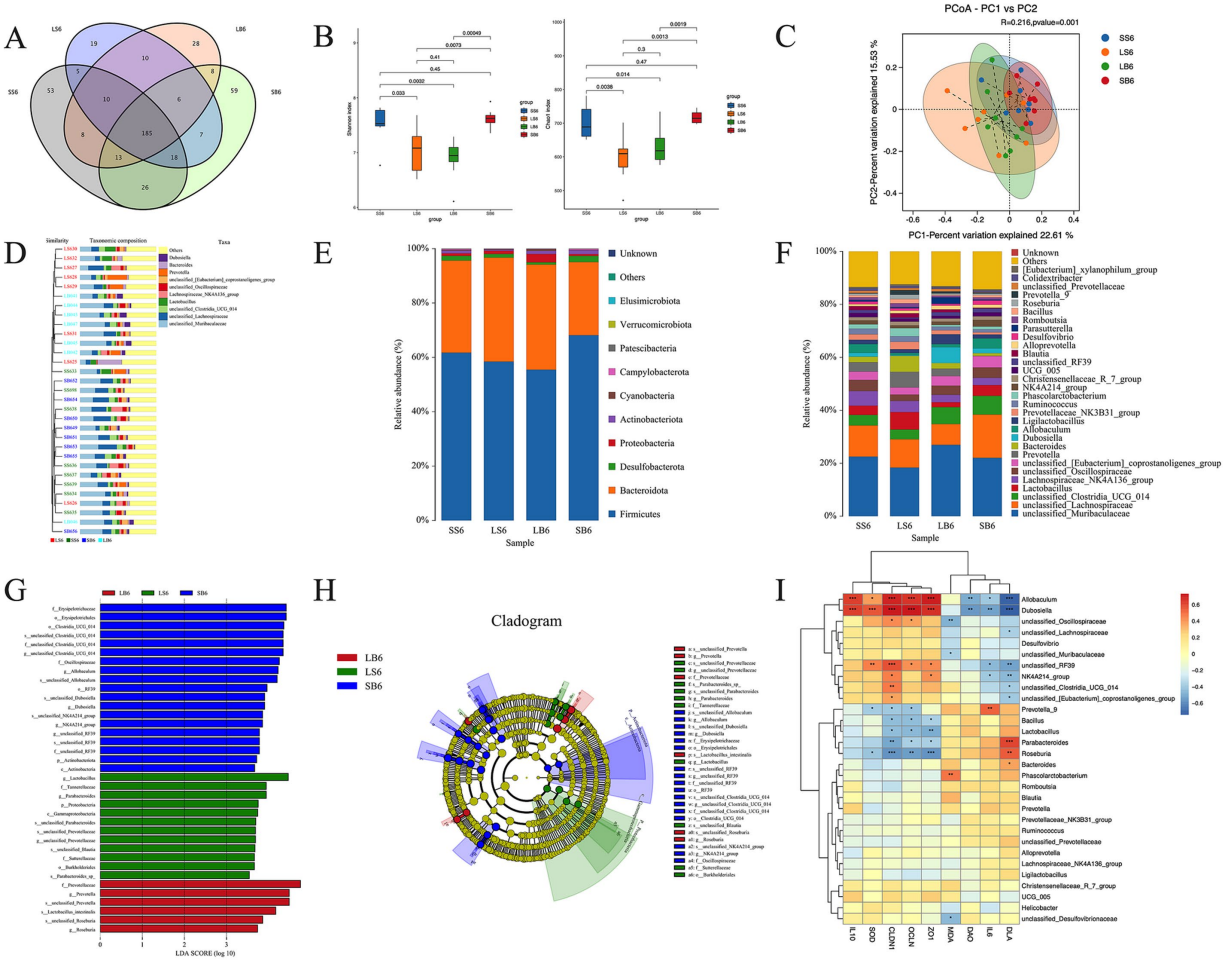
Effect of *Bifidobacterium longum* JBLC-141 on hypoxic intestinal flora of rats under simulated plateau conditions. **(A)** Venn diagram. **(B)** Chao1 and Shannon indexes. **(C)** Principal coordinates analysis (PCoA). **(D)** Unweighted pair group method with arithmetic mean (UPGMA) analysis of bacterial communities. Relative abundance of **(E)** phyla and **(F)** genera. **(G)** Linear discriminant analysis (LDA) scores. **(H)** Taxonomic cladogram of LDA effect size (LefSe) analysis. **(I)** Correlation analysis of intestinal flora with oxidative stress index, inflammatory factor, intestinal compact connection index, and intestinal permeability index. *p < 0.05, **p < 0.01, ***p < 0.001, and ****p < 0.0001.

We further investigated the correlation between the intestinal flora composition and barrier damage in each experimental group. The Venn diagrams showed that 455 genera were identified in all samples, with 318, 260, 265, and 322 specific genera identified in the SS, LS, LB, and SB groups, respectively. Among the four groups, 185 genera were identified, with 53, 19, 28, and 59 unique to the SS, LS, LB, and SB groups, respectively (Figure 4A). Hence, *B. longum* JBLC-141 had a regulatory effect on the intestinal flora of rats in the low-oxygen environment of the plateau.

Microbial diversity analysis showed that the Shannon and Chao indices of the LS group were lower than those of the SS group, indicating that plateau hypoxia decreased the diversity and abundance of the intestinal flora. However, no significant difference was observed between the LB and LS groups (Figure 4B). PCoA and UPGMA analyses of bacterial communities showed a significant separation between the LS group and the SS and SB groups. Therefore, plateau hypoxia decreased changes in the structure of intestinal bacterial communities. Meanwhile, JBLC-141 pretreatment relatively restored the structure to that of the SS and SB groups (Figures 4C,D).

Analysis of the microbial phyla composition identified Firmicutes, Bacteroidetes, Desulfobacterota, Proteobacteria, and Actinobacteria as the major phyla in each group, accounting for >99% of the total microbial population. The relative Actinobacteria population in the LS group was reduced but recovered after JBLC-141 pretreatment (Figure 4E). Analysis of the top 20 phylogenetic compositions showed that the LS group contained unclassified_Muribaculaceae, unclassified_Clostridia_UCG-014, unclassified_Oscillospiraceae, unclassified_Eubacterium_coprostanoligenes_group, *Dubosiella*, *Allobaculum*, *Ligilactobacillus*, and *NK4A214*_*group*. Furthermore, this group exhibited a reduced and increased abundance of *Lactobacillus*, *Prevotella*, *Bacteroides*, *Prevotellaceae*_*NK3B31*_*group*, *Phascolarctobacterium*, Christensenellaceae_R_7 group, and *Blautia*. Notably, JBLC-141 pretreatment of the LB group reversed these trends (Figure 4F). Biomarkers that differed significantly between the groups were identified. Further analysis of the rat intestinal flora in each group using LEfSe showed that g_*Lactobacillus*, f_Tannerellaceae, g_*Parabacteroides*, p_Proteobacteria, c_Gammaproteobacteria, s_unclassified_*Parabacteroides*, s_unclassified_Prevotellaceae, g_unclassified_Prevotellaceae, s_unclassified_*Blautia*, f_Sutterellaceae, o_Burkholderiales, and s_*Parabacteroides*_spp were significantly enriched in the LB group. In contrast, f_Prevotellaceae, g_*Prevotella*, s_Lactobacillus_*intestinalis*, s_unclassified_*Prevotella*, s_unclassified_*Roseburia*, and g_*Roseburia* were significantly enriched in the LS group (Figures 4G,H).

Correlation analysis was conducted using hierarchical clustering based on Spearman’s correlation of differential flora using oxidative stress-related and intestinal barrier function indicators. At the genus level, the serum pro-inflammatory factor, IL-6, and the indicator of intestinal barrier permeability, DLA, were significantly negatively correlated with *Allobaculum*, *Dubosiella*, unclassified_RF39, and *NK4A214_group*. In addition, IL-6 was significantly and positively correlated with *Prevotella*_*9*, whereas DLA was significantly and positively correlated with unclassified_*Clostridia*_*UCG-014*. DLA was also negatively correlated with *unclassified_Clostridia_UCG_014*, *unclassified_[Eubacterium]_coprostanoligenes_group*, and significantly positively correlated with *Parabacteroides*, *Roseburia*, and *Bacteroides*.

SOD, which removes reactive oxygen radicals from colon tissue, and colon tissue tight junction proteins ZO-1, OCLN, and CLDN1 were significantly and positively correlated with *Allobaculum*, *Dubosiella*, and *unclassified_RF39*. Intestinal tissue ZO-1, OCLN, and CLDN1 were negatively correlated with *Bacillus*, *Lactobacillus*, *Parabacteroides,* and *Roseburia*. The colonic tissue oxidative stress product, MDA, content was negatively correlated with *unclassified_Oscillospiraceae*, *unclassified_Muribaculaceae*, and *unclassified_Desulfovibrionaceae* and positively correlated with *Phascolarctobacterium*.

## 4 Discussion

In this study, we elucidated the mechanism underlying the mitigating effects of *B. longum* JBLC-141 on intestinal barrier damage caused by plateau low-pressure hypoxia. Intestinal damage accompanied by dysbiosis of the intestinal flora was successfully induced in the rat model by exposure to a hypobaric chamber. Consistent with previous studies, the epithelial structure and microbial communities of the established model were significantly altered under hypoxia. In particular, high-altitude hypoxia alters the diversity of gut microbiota in rats or mice (Ma et al., 2023; Wan et al., 2022). Previous studies have emphasized the critical role of cuprocytes in regulating the intestinal barrier in healthy and diseased states (Bergstrom et al., 2020; Birchenough et al., 2016; Grondin et al., 2020).

In the current study, exposure to low-pressure hypoxia on a simulated plateau significantly reduced the number of cuprocytes in intestinal areas of the rat model. This will decrease intestinal mucus secretion, resulting in thinning of the intestinal mucus layer and increased permeability of the intestinal barrier (Gustafsson and Johansson, 2022). Additionally, the intestinal barrier function depends on maintaining tight junction proteins, such as ZO-1, OCLN, and CLDN, which are crucial to forming the mechanical barrier of the mucosa. Accordingly, these tight junction proteins are important markers of intestinal barrier integrity. Consistent with previous findings (Cheng et al., 2022), exposure to simulated plateau hypoxic conditions decreased ZO-1 and OCLN expression levels in the current rat model. Under normal conditions, the intestinal mucosa exhibits selective permeability that allows beneficial substances to enter the body while preventing the passage of harmful substances. However, increased intestinal permeability creates more opportunities for endotoxins and pathogens to traverse the tissue, increasing the risk of infection and disease.

Moreover, increased intestinal permeability increases the serum DAO and DLA content, which can indirectly respond to changes in intestinal permeability. DAO, as an intracellular enzyme with high activity in the villi of the small intestinal mucosa, is an important indicator of mechanical barrier integrity (Tao et al., 2022; Zhou et al., 2011). DAO produced by intestinal mucosal cells and DLA—a metabolite of intestinal bacteria—can enter the bloodstream when the intestinal barrier is damaged (Cai et al., 2019; Song et al., 2009). Therefore, serum levels of these two proteins can reflect the degree of intestinal barrier damage (Cai et al., 2019; Song et al., 2009). In this study, JBLC-141 reduced the serum levels of DLA and DAO induced by the simulated plateau low-pressure hypoxia in rats. This indirectly indicates that probiotic JBLC-141 exerts a protective role.

Furthermore, *B. longum* JBLC-141 pretreatment effectively reduced the intestinal inflammatory response and barrier damage induced by plateau hypoxia, promoted the expression of intestinal intercellular tight junction proteins, and reduced intestinal permeability. These findings are similar to those of Khanna et al. (2019), who studied acute hypoxic exposure in rats. They found different degrees of intestinal mucosal damage that manifested as increased mucosal permeability and damage to the structure of intestinal villi. The entry of harmful substances produced by the intestinal flora into the bloodstream triggers a local or systemic inflammatory response (Wang et al., 2018), a critical factor responsible for disrupting intestinal barrier function (Tao et al., 2022). Elevated pro-inflammatory cytokine levels (e.g., IL-1β, IL-6, and TNF-*α*) disrupt tight junctions, creating a cycle that leads to further deterioration of the intestinal barrier function and exacerbation of intestinal inflammation (Kaminsky et al., 2021; Zhang et al., 2023).

Various probiotics, such as *B. longum*, can alleviate the intestinal inflammatory response by inhibiting the expression of pro-inflammatory cytokines while promoting the inhibitory factor IL-10 (Cao et al., 2023; Tang et al., 2024). In the present study, pretreatment with JBLC-141 significantly downregulated IL-6 and upregulated IL-10. This effect improved intestinal barrier damage, suggesting that JBLC-141 pretreatment attenuated the intestinal barrier damage and inflammatory response induced by the simulated plateau hypoxia.

Oxidative stress is induced by low-pressure hypoxia on plateaus (Hou et al., 2024; Zhang et al., 2022b). Under these conditions, intestinal cell mitochondria produce a large amount of ROS, the accumulation of which leads to mitochondrial structural damage. Moreover, ROS can penetrate the mitochondrial membrane and enter the cytoplasm, damaging intracellular macromolecules (Hou et al., 2024). In the mitochondrial damage pathway, the anti-apoptotic BCL-2 induces the release of cytochrome c into the mitochondrial membrane, mediating the activation of the pro-apoptotic protein CASP3 (Ulukaya et al., 2011). Low-pressure hypoxia induced apoptotic shedding of mucosal cells in the intestinal epithelium. Meanwhile, JBLC-141 decreased the abundance of CASP3 and increased that of BCL-2. Thus, *B. longum* JBLC-141 effectively alleviated intestinal tissue cell apoptosis in plateau hypoxic rats.

In addition to inhibiting ROS-induced apoptosis of intestinal epithelial cells*, B. longum* increased the expression of SOD while inhibiting MDA production. Previous studies have shown that *B. longum* attenuates oxidative stress damage. In the current study, the plateau hypoxia-induced changes in oxidative stress markers in the gut barrier were attenuated by JBLC-141. Similarly, *B. longum* significantly reduced and increased MDA and SOD liver concentrations in a mouse model of alcoholic liver disease, attenuating alcohol-induced oxidative stress in the liver (Ulukaya et al., 2011). JBLC-141 pretreatment also downregulated KEAP1 and upregulated NRF2 and HO-1 levels. This is consistent with a previous study exploring the protective effect of *B. longum* against intestinal barrier damage caused by intestinal ischemia–reperfusion (Tang et al., 2024). The NRF2–HO1 pathway is an important endogenous antioxidant system that attenuates ROS-induced oxidative stress (Vasileva et al., 2020). Therefore, we hypothesize that KEAP1–NRF2–HO1 likely mediates the mechanisms by which JBLC-141 attenuates oxidative stress injury in the intestinal tract of rats in the plateau low-pressure, low-oxygen environment.

Low-pressure hypoxia in highland areas alters the species composition and metabolism of intestinal flora (Jia et al., 2020; Wu et al., 2020). In this study, the LS group exhibited a decreased relative abundance of genera of beneficial bacteria compared to the SS group feces samples. These changes may reflect intestinal damage caused by inflammation and oxidative stress damage in intestinal epithelial cells in rats exposed to high altitudes. However, pretreatment with *B. longum* JBLC-141 reversed these effects. Most beneficial flora are producers of SCFAs, which exert anti-inflammatory effects by modulating cytokine production and immune cell function (Liu et al., 2021). SCFAs may inhibit oxidative stress by regulating oxidoreductase activity (Liu et al., 2021). The Muribaculaceae family participates in forming the mucus layer and barrier function in the colon (Volk et al., 2019) and hypoxic stress adaptation and inflammatory response (Zhang et al., 2022a). The unclassified Oscillospiraceae in the phylum with thick-walled bacteria are key in dietary fiber degradation and are thought to be butyric acid producers (Konikoff and Gophna, 2016); they may also use the host polysaccharides as a substrate for growth to alleviate obesity-induced chronic inflammation (Konikoff and Gophna, 2016).

*Dubosiella* relative abundance positively correlates with GSH-Px, T-AOC, SOD, NRF-2, HO-1, and IL-10 levels and negatively correlates with MDA, IL-1β, IL-6, and TNF-*α* levels (Ai et al., 2021; Wan et al., 2021a; Wan et al., 2021b). Therefore, *Dubosiella* spp. enhance the antioxidant and anti-inflammatory capacity of the organism, consistent with Spearman’s correlation analysis in the current study. Additionally, *Dubosiella* exerts antiaging effects by attenuating oxidative stress, enhancing vascular endothelial function, and balancing the intestinal flora. Moreover, *Dubosiella* and its metabolite butyric acid inhibit microglia-mediated inflammation and oxidative stress and protect neurons from LPS-induced damage through the G protein-coupled receptor 109A (GPR109A)/NRF2/HO-1 pathway (Zhang H. et al., 2022).

*Allobaculum* enhances the intestinal antioxidant capacity by producing SCFAs (Yi et al., 2023). The long-chain fatty acid C18-3OH produced by *Allobaculum* acts as an agonist of peroxisome proliferator-activated receptor *γ*, which may contribute to its anti-inflammatory properties (Pujo et al., 2021). This antioxidant effect can be activated by increasing the activity of enzymes, such as SOD and CAT, and by the function of the peroxisome family. *Allobaculum* was positively correlated with intestinal tight junction proteins, IL-10, and SOD and negatively correlated with IL-6, DLA, and DAO. Furthermore, *Allobaculum* exhibited antioxidative stress and anti-inflammatory activities while enhancing the intestinal barrier function. However, further studies to determine whether these effects of *Allobaculum* require the activity of other metabolites are warranted. These microbial species could serve as biomarkers in determining the effectiveness of *B. longum* JBLC-141 in plateau hypoxic intestinal barrier damage, warranting further investigation. RF39 inhibits *Escherichia coli* overgrowth, negatively correlates with LPS biosynthesis (Ali et al., 2022), and exerts antioxidant and anti-inflammatory effects (Chen et al., 2024).

In conclusion, low-pressure plateau hypoxia can alter the composition of the gut microbiota, and these changes may exacerbate intestinal injury. Therefore, strategies for the prevention and treatment of plateau hypobaric hypoxia-induced intestinal barrier dysfunction must consider effects on the gut flora. *B. longum* JBLC-141 pretreatment can improve the beneficial microbial composition by interfering with the intestinal flora, and can effectively construct a biological barrier to attenuate plateau hypobaric hypoxia-induced damage to the intestinal barrier.

Although this study found that *B. longum* JBLC-141 can counteract intestinal oxidative stress, inhibit inflammatory responses, and improve intestinal permeability and apoptosis, our findings are only relevant. Whether the improvement of oxidative stress by *B. longum* JBLC-141 is related to the inhibition of inflammatory responses and the interrelationships between intestinal inflammatory responses and intestinal oxidative stress need to be further clarified. In addition, whether the protective effect of *B. longum* JBLC-141 on the intestinal barrier is related to changes in the metabolites of the intestinal flora needs to be further explored. In the follow-up study, we will investigate the effects of *B. longum* JBLC-141 on the metabolites of intestinal flora by metabolomics in an attempt to find the possible mechanisms by which the flora metabolites are involved in the amelioration of intestinal inflammatory response and intestinal oxidative stress injury.

## 5 Conclusion

This study demonstrated that *B. longum* JBLC-141 counteracts intestinal oxidative stress, inhibits inflammatory responses, improves intestinal permeability and apoptosis, and upregulates the expression of intestinal tight junction proteins. These effects are likely mediated by promoting the KEAP1/NRF2/HO1 signaling pathway and simultaneously inhibiting the intestinal barrier damage. In addition, *B. longum* JBLC-141 regulates changes in the intestinal flora, which ultimately promotes the intestinal function of rats under low-pressure, low-oxygen conditions in the simulated plateau environment. Therefore, the protective effect of JBLC-141 against plateau hypoxia-induced intestinal tissue damage may be mediated through its effect on intestinal flora composition.

## Data Availability

The 16s rRNAseq data presented in the study are deposited in the NCBI database under the Bioproject, accession number PRJNA1189695.
