# Supplementary material for: Bifidobacterium longum JBLC-141 alleviates hypobaric hypoxia-induced intestinal barrier damage by attenuating inflammatory responses and oxidative stress
Source: Front Microbiol. 2024 Dec 17;15:1501999. doi: 10.3389/fmicb.2024.1501999 (PMC11685222; doi:10.3389/fmicb.2024.1501999)
Supplement: Supplementary file 1 [file Table_1.docx]

Table S1 WB antibody information table

| Detection antibody | molecular weight | Primary antibody | Secondary antibody |
| --- | --- | --- | --- |
| β-actin | 42 kDa | Servicebio GB15003 1: 5000 | Servicebio HRP-Goat Anti-Rabbit  GB23303 1：5000 |
| ZO-1 | 225 kDa | Thermofisher 40-2200 1: 1000 | Servicebio HRP-Goat Anti-Rabbit  GB23303 1：5000 |
| Occludin | 52 kDa | Thermofisher 33-1500 1: 1000 | Servicebio HRP-Goat Anti-Mouse  GB23301 1：5000 |
| Claudin-1 | 19 kDa | Abcam ab307692 1: 1000 | Servicebio HRP-Goat Anti-Rabbit  GB23303 1：5000 |
| Keap1 | 60 kDa | Servicebio GB113747 1:1000 | Servicebio HRP-Goat Anti-Rabbit  GB23303 1：5000 |
| Nrf2 | 90-100 kDa | Servicebio GB115673 1:1000 | Servicebio HRP-Goat Anti-Rabbit  GB23303 1：5000 |
| HO-1 | 33 kDa | Servicebio GB115713 1:1000 | Servicebio HRP-Goat Anti-Rabbit  GB23303 1：5000 |
| c-caspase3 | 17 kDa | Affinity AF7022 1:1000 | Servicebio HRP-Goat Anti-Rabbit  GB23303 1：5000 |
| Bcl2 | 26 kDa | Servicebio GB154380 1:1000 | Servicebio HRP-Goat Anti-Rabbit  GB23303 1：5000 |
| Lamin B1 | 66-70 kDa | Servicebio GB115715 1: 5000 | Servicebio HRP-Goat Anti-Rabbit  GB23303 1：5000 |
